# Potential Clinical Value of Biomarker-Guided Emergency Triage for Thoracic Aortic Dissection

**DOI:** 10.3389/fcvm.2021.777327

**Published:** 2022-01-12

**Authors:** Peng Qiu, Meng Yang, Hongji Pu, Jingli Hou, Xu Chen, Zhaoyu Wu, Qun Huang, Siyi Huang, Yan Fu, Zi'ang Wen, Chengxin Zhang, Binshan Zha, Yang Yang, Zhijue Xu, Fuxiang Chen, Xinwu Lu

**Affiliations:** ^1^Department of Vascular Surgery, Shanghai Ninth People's Hospital, Shanghai Jiao Tong University School of Medicine, Shanghai, China; ^2^Department of Clinical Laboratory, Shanghai Ninth People's Hospital, Shanghai Jiao Tong University School of Medicine, Shanghai, China; ^3^Instrumental Analysis Center, Shanghai Jiao Tong University, Shanghai, China; ^4^Department of Cardiovascular Surgery, Department of Vascular and Thyroid Surgery, Department of General Surgery, The First Affiliated Hospital of Anhui Medical University, Hefei, China; ^5^Department of Computer Science and Engineering, Shanghai Jiao Tong University, Shanghai, China; ^6^Shanghai Key Laboratory of Tissue Engineering, Shanghai Ninth People's Hospital, Shanghai JiaoTong University School of Medicine, Shanghai, China; ^7^Vascular Center of Shanghai JiaoTong University, Shanghai, China

**Keywords:** thoracic aortic dissection, emergency triage, biomarker, proteomics, serum

## Abstract

**Aim:** Thoracic aortic dissection (TAD) is a high-risk vascular disease. The mortality rate of untreated TADs in 24 h was as high as 50%. Thus, rapid diagnosis of TAD in the emergency department would get patients to the right treatments to save their lives.

**Methods:** We profiled the proteome of aortic tissues from TAD patients using a label-free quantification proteomics method. The differentially expressed proteins were screened and subjected to bioinformatics analysis. Candidate biomarkers were selected and validated in independent serum samples using enzyme-linked immunosorbent assays (ELISAs). The diagnostic values were further predicted *via* receiver operating characteristic (ROC) curve analysis.

**Results:** A total of 1,141 differentially expressed proteins were identified in aortic tissues from 17 TAD patients and eight myocardial infarction (MI) patients. Six proteins were selected as candidate biomarkers for ELISAs in an independent training set of 20 serum samples (TAD = 10, MI = 10). Of these proteins, four with a *P*-value < 0.01 were further validated in another independent set of 64 serum samples (TAD = 32, MI = 32) *via* ELISAs. ITGA2, COL2A1, and MIF had *P*-values < 0.0001, and their areas under the curve (AUCs) were 0.801 (95% CI: 0.691–0.911), 0.773 (95% CI: 0.660–0.887), and 0.701 (95% CI: 0.574–0.828), respectively.

**Conclusion:** ITGA2, COL2A1, and MIF were identified as promising biomarkers for discriminating TAD from emergency patients with severe chest pain. Biomarker-guided emergency triage could further shorten the time for patients to get more effective treatments.

## Introduction

Thoracic aortic dissection (TAD) is an emergency characterized by the rapid development of an intimal flap resulting from the flushing of blood flow between the intima and the adventitia after rupture of the thoracic aortic intima induced by mechanical factors (1, 2). As one of the most common lethal aortic diseases, ~20% of patients with TADs die before onset or diagnosis. For untreated TADs, the mortality rate is ~25% at 6 h and 50% by 24 h. TAD pathology is characterized primarily by lumen dilatation and medial degeneration with vascular smooth muscle cell (VSMC) and extracellular matrix (ECM) abnormalities, resulting in abnormal tissue biomechanics and progressive disease (3). TAD is rapidly fatal due to aortic rupture, organ ischemia, or cardiac tamponade. According to whether the ascending aorta and the aortic arch are involved, the dissection can be divided into Stanford type A and type B. Taking type A as an example, global data [International Registry of Aortic Dissection (IRAD)] indicate that the in-hospital mortality rate is as high as 13–22%. Even though breakthrough advances in surgical techniques, levels of surveillance, and treatment strategies have occurred in the last 20 years, the mortality rate remains high (4).

TAD has the same symptoms as many other more common diseases including myocardial infarction (MI), vascular embolization, gastric ulcer, or acute back pain (5). As many as 40% of TAD cases may be missed, misdiagnosed, or overlooked in the emergency room, and sometimes can only be identified at autopsy (6). The difficulty in completing the diagnostic process of TAD, such an emergency that needs to be controlled rapidly, may lead to the delay of management (7). According to IRAD investigators, the meantime to treatment or at least definitive diagnosis is more than 6 h in Europe and exceeds 15 h in the USA (8). In China, according to our experience, it often takes a day or more for a TAD patient in a rural area to enter the emergency department of the specialized hospital. TAD and MI are two of the most serious diseases that present with severe chest pain, but as mentioned, the two diseases are difficult to distinguish in the emergency department due to the inaccessible diagnostic examination for TAD (9). In China, only tertiary hospitals have CT angiography and experienced radiologists to identify TADs. In addition, there is no widely available, rapid non-invasive biomarker to distinguish TAD from other high-risk chest pain in clinical practice (10). Similar to myocardial injury markers (cardiac troponin, cTn; and creatine kinase-MB, CK-MB) and cardiac function markers (brain natriuretic peptide, BNP; and pro-BNP) for MI, and D-dimer for pulmonary embolism, we hope to identify a biomarker specific to aortic dissection in the case of severe chest pain.

Aortic dissection repair (including open surgery and endovascular aortic repair) only physically excludes the proximal segment of the dissected vessels, without completing biological healing. The aorta, especially the distal part of the covered stent, remained in the pathologic state of dissection (11, 12). Postoperative healing after complete false lumen thrombosis occurred in some patients, and recurrence of dissections occurred in others (13). Therefore, systematic analysis of the biological alterations in the aorta of TAD patients might provide potential therapeutic targets for postoperative TAD therapy.

For the past few years, proteomics technology has been rapidly developed and widely applied in biomedical studies along with the great evolution of mass spectrometry (MS) methodology (14–17). The sensitivity and repeatability of MS have been powerful enough for the comprehensive profiling of proteomes from clinical tissue samples and deep screening of valuable biomarkers for disease diagnosis (18–20). In this study, we aimed to identify differentially expressed proteins in crevasse tissues from TAD patients and to screen and validate potential biomarkers of TAD in independent serum samples. Moreover, based on the tissue proteome of TAD, we described the possible mechanisms involved and provided potential therapeutic targets for improving the prognosis of aortic dissection.

## Materials and Methods

### Study Design

**Figure 1:**
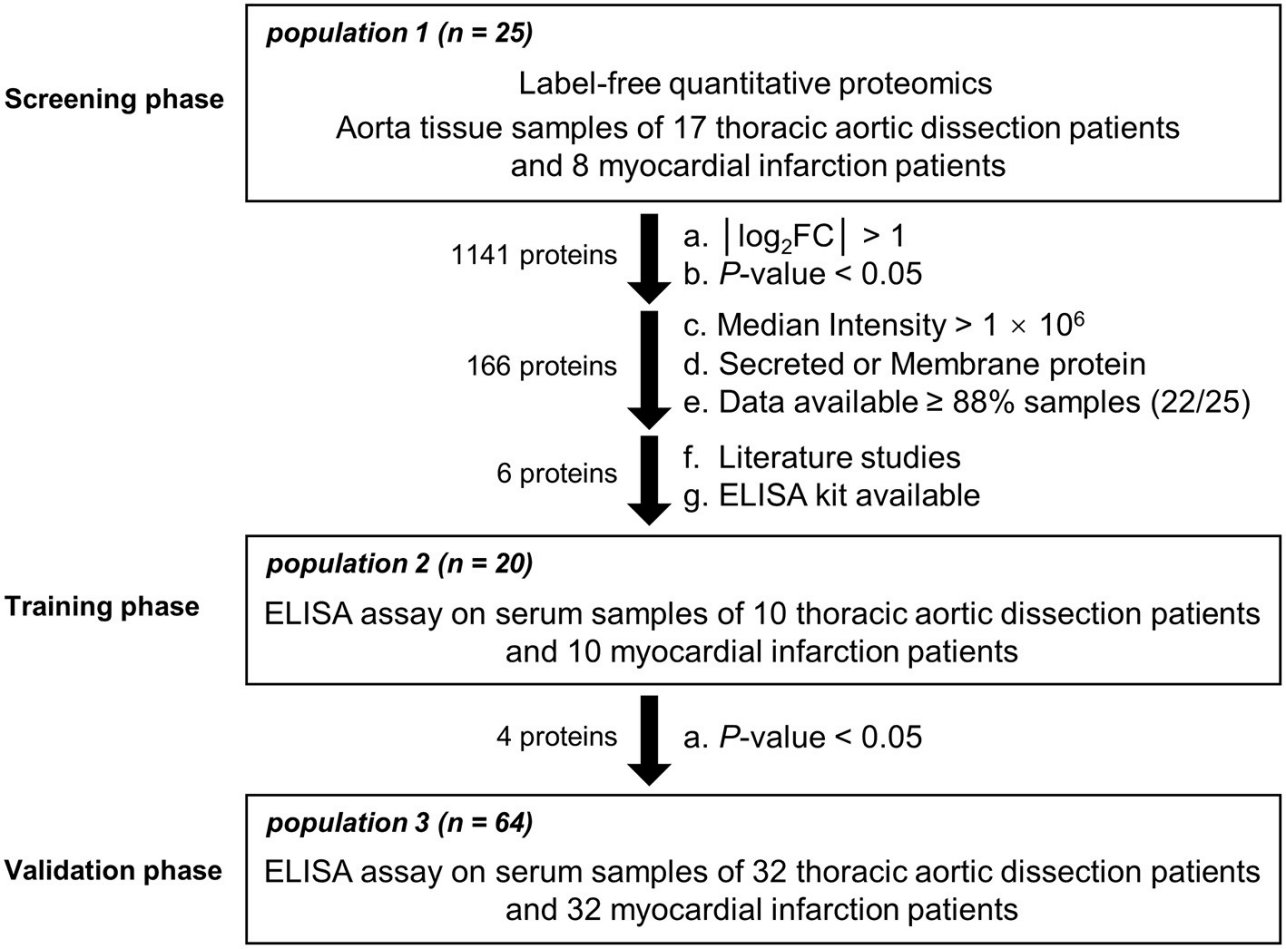
Flowchart of the study design. Participants from 3 different populations were entered into the screening, training, and validation phases.

The present study consisted of three phases (Figure 1). Phase 1 was the screening phase in which aortic tissue samples from 17 TAD patients and 8 MI patients with atherosclerosis undergoing coronary artery bypass grafting (CABG) without aortic dilatation (population 1) were collected. During the CABG, the ascending aorta wall with a diameter of about 5–7 mm was cut, followed by anastomosing a saphenous vein graft to the ascending aorta (21). All the samples were subjected to label-free quantitative proteomics analysis to explore differentially expressed proteins. Proteins with the fold changes (FC) >2 or <0.5 and a *P*-value < 0.05 were identified as differentially expressed. Proteins with a median intensity >1 × 10^6^, a subcellular location of secreted or the cell membrane (22), and that were detected at least in 22 samples were subjected to a literature review to select candidates for the training phase. Phase 2 was the training phase in which serum samples from 10 TAD patients and 10 MI patients (population 2) were collected and subjected to enzyme-linked immunosorbent assays (ELISAs). Proteins with a *P*-value < 0.05 were identified as candidates for the validation phase. Phase 3 was the validation phase in which serum samples from 32 TAD patients and 32 MI patients (population 3) were collected and subjected to ELISAs.

### Subjects

All TAD patients satisfied the following inclusion criteria: (a) TAD was confirmed by aortic computed tomography angiography (CTA) or angiography; (b) age over 18 years; (c) within 14 days after the onset of symptoms; (d) without traumatic dissections or inflammatory dissections; (e) without other aortic diseases, such as aneurysms; and (f) not pregnant. All MI patients satisfied the following inclusion criteria: (a) MI was confirmed by coronary artery angiography, (b) age over 18 years, and (c) confirmed by CTA to have atherosclerosis without aortic dilatation. More details can be found in the Supplementary Materials.

According to the inclusion and exclusion criteria, all subjects enrolled in the screening phase were obtained from the First Affiliated Hospital of Anhui Medical University in the region (2019.10–2020.06). In addition, patients in the independent training set and validation set were recruited from Shanghai Ninth People's Hospital, Shanghai Jiao Tong University, School of Medicine in the region (2020.09–2021.06).

### Ethics Statement

This study was carried out in accordance with the principles of the Declaration of Helsinki and was approved by the Ethics Committee of Shanghai Ninth People's Hospital, Shanghai Jiao Tong University, School of Medicine, and the First Affiliated Hospital of Anhui Medical University (approval ID: SH9H-2019-T144-2). Written informed consent was obtained from all participants.

### Label-Free Quantitative Proteomics Analysis

All tissues were lysed in UA solution (8 M urea in 0.1 M Tris-HCl) containing protease inhibitors (Thermo Fisher), followed by mechanical grinding at a frequency of 60 Hz at 4°C for 3 min. The lysate was centrifuged at 15,000 g for 10 min, and the supernatant was collected as a whole extract. The protein concentrations were determined using the BCA assay (Thermo Fisher). Samples were then digested using the filter-aided sample preparation method (23). Extracts of 100 μg protein from each sample were reduced with 10 mM dithiothreitol at 37°C for 1 h and then alkylated with 50 mM iodoacetamide at room temperature in the dark for an additional 40 min. The sample solution was centrifuged on a 10-kDa filter at 14,000 g for 20 min, followed by washing with 200 μl of UA solution three times and 200 μl of 50 mM ammonium bicarbonate three times by centrifugation at 14,000 g for 20 min. Then, 100 μl of 50 mM ammonium bicarbonate with 2 μg trypsin was added. The tubes were incubated at 37°C for 12 h. The peptides were collected by centrifugation at 14,000 g for 20 min. The filters were washed three times with 50 μl of 50 mM ammonium bicarbonate by centrifugation at 14,000 g for 20 min. All flow-through fractions were collected and pooled. The peptides were desalted on C18 ZipTips (Millipore, Billerica, MA, USA), freeze-dried and then stored at −80°C before use.

Liquid chromatography tandem MS (LC-MS/MS) analysis was performed by coupling a nanoLC column (Easy nLC1200, Thermo Fisher Scientific) to an Orbitrap Q-Exactive Plus mass spectrometer (Thermo Fisher Scientific). In brief, 1.0 μg of peptide mixture from each sample was dissolved in buffer A (0.1% formic acid in water), delivered to an analytical column (Dikma, inspire C18, 3 μm, Canada, 200 mm × 50 μm) and separated over a 120-min gradient at a flow rate of 0.3 μL/min as follows: 0–3 min 2–6% B, 3–95 min 6–20% B; 95–107 min 20–32% B; 107–108 min 32–100% B; and 108–120 100–100% B (buffer B, 80% acetonitrile containing 0.1% formic acid). MS analysis was performed using MS1 scans (350–1,500 m/z) for intact peptides acquired on the Orbitrap at 70,000 resolution and an automatic gain control target of 3e6 ions, followed by MS2 scans of up to 20 abundant multiply charged precursors in the MS1 spectrum fragmented by the higher energy collisional dissociation (HCD) with a normalized collision energy of 28 on the Orbitrap.

The raw data were processed using Proteome Discoverer 2.4 software (PD 2.4, Thermo Fisher Scientific) and searched against the human-specific UniProt KB-reviewed database containing 20,286 entries (downloaded on November 11, 2020). The mass tolerances were 10 ppm for precursor ions and 0.02 Da for product ions. Up to two missed cleavages were allowed. Carbamidomethylation on cysteine residues was used as a fixed modification; N-acetylation and oxidation of methionine were used as variable modifications. The false discovery rates were set to <1%.

### Proteome Data Analysis

Principal component analysis (PCA) was performed using the R package factoextra. A correlation heatmap was generated using the R package corrplot (24). Functional annotation clustering of differentially expressed proteins was performed using DAVID 6.8 (https://david.ncifcrf.gov/) (25, 26). The enrichment analysis of differentially expressed proteins was implemented with the R package clusterProfiler based on the Gene Ontology (GO, http://geneontology.org/) and Kyoto Encyclopedia of Genes and Genomes (KEGG, http://www.kegg.jp/) databases (27, 28). The protein–protein interaction networks were constructed using STRING (https://www.string-db.org/) (29) and visualized with Cytoscape 3.8.2 (30). The functional clusters were defined via the Molecular Complex DEtection (MCODE) plugin (31). Heatmaps were drawn using the R package ComplexHeatmap with the hierarchical clustering method. Gene set enrichment analysis was performed using GSEA 4.1.0 (32).

### ELISAs

Integrin alpha-2 (ITGA2), macrophage migration inhibitory factor (MIF), Parkinson's disease protein 7 (PARK7), microfibrillar-associated protein 5 (MFAP5), junctional adhesion molecule A (JAMA), and collagen alpha-1 II (COL2A1) were subjected to ELISAs following the manufacturer's recommendations. The ITGA2, MFAP5, JAM-A, and PARK7 kits were purchased from Reybiotech (Heidelberg, Germany), the COL2A1 kit was purchased from USCN (Wuhan, China), and the MIF kit was purchased from BOSTER. Each sample was tested in duplicate (Wuhan, China).

### Statistical Analysis

Statistical analyses of population characteristics and protein expression levels were performed using GraphPad Prism 5.0 software (San Diego, CA, USA). Comparisons between two groups were performed using Student's *t*-tests (two–tailed) or the Mann-Whitney *U*-test. The area under the receiver operating characteristic (ROC) curve (AUC), sensitivity, and specificity were calculated using SPSS 20.0 statistical software (IBM, New York, NY, USA). In the validation phase, the predicted values of the combination of each protein were calculated by binary logistic regression using SPSS 20.0 statistical software (IBM, New York, NY, USA).

## Results

### Identification of Differentially Expressed Proteins

**Figure 2:**
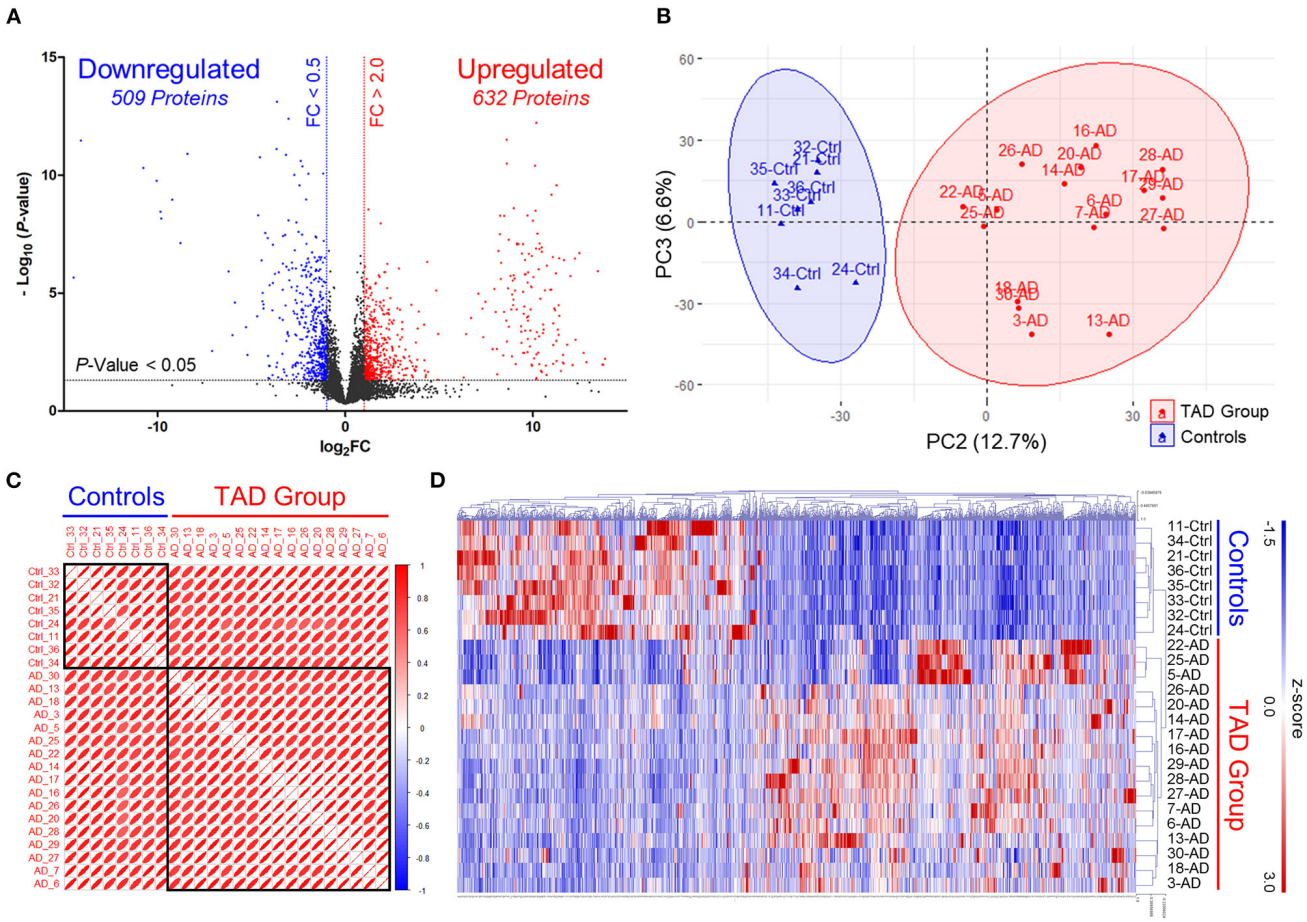
Discovery of differentially expressed proteins *via* label-free quantitative proteomics profiling. **(A)** Volcano plots illustrating differentially expressed proteins. Red circles, significantly upregulated proteins in TAD samples. Blue circles, significantly downregulated proteins in TAD samples. Control, MI samples. FC, expression ratios of TAD/Control. **(B)** Principal component analysis (PCA) plots separate TAD and control samples. Scatter plots show PC2 against PC3. The scatter plots of PC1 against PC2 and PC1 against PC3 are shown in Supplementary Figure 1. **(C)** Correlation heatmap shows Pearson's correlations between all TAD and control samples. Red boxes are grouped by clusters (ward.D). **(D)** Cluster heatmap of the 632 upregulated and 509 downregulated proteins in TAD samples. Samples are clustered according to Pearson's correlations.

At the screening phase, 25 aortic tissue samples from the TAD (*n* = 17) and control (*n* = 8) groups were subjected to label-free quantitative proteomics profiling. The clinical characteristics of the TAD and control groups are summarized in Table 1. Patients in the TAD group were younger than patients in the control group (53.35 ± 13.22 vs. 68.38 ± 7.27 years, *P*-value = 0.007). There was no significant difference in sex, BMI, hypertension, diabetes, neurological status, or smoking distribution between the two groups. No patients with a bicuspid aortic valve or Marfan syndrome were included in our study. A total of 6,045 proteins were identified, of which 632 were upregulated (FC of TAD/Control >2.0, *P*-value < 0.05) and 509 were downregulated (FC of TAD/Control <0.5, *P*-value < 0.05) in TAD (Figure 2A). Samples from the two groups were obviously separated by both PCA and correlation heatmap clustering (Figures 2B–D), indicating that the proteomes of the aortic tissues from the TAD and control groups were significantly different.

**Table 1:** Characteristics of the TAD group and control group in the screening phase.

**Variable**	**TAD (*n* = 17)**	**Control (*n* = 8)**	***P*-value**
Age (years)	53.35 ± 13.22	68.38 ± 7.27	0.007
Male	11 (64.7%)	5 (62.5%)	0.915
BMI (kg/m^2^)	23.66 ± 2.54	23.86 ± 2.89	0.797
Hypertension	14 (82.4%)	5 (62.5%)	0.278
Diabetes	1 (5.9%)	1 (12.5%)	0.569
Smoker	10 (58.8%)	5 (62.5%)	0.861
Marfan syndrome	0	0	1.000
Bicuspid aortic valve	0	0	1.000

*Control group: tissue samples from MI patients. BMI, Body mass index*.

### Bioinformatics Analysis Reveals the Downregulation of ECM and Collagen Organizations in TAD

**Figure 3:**
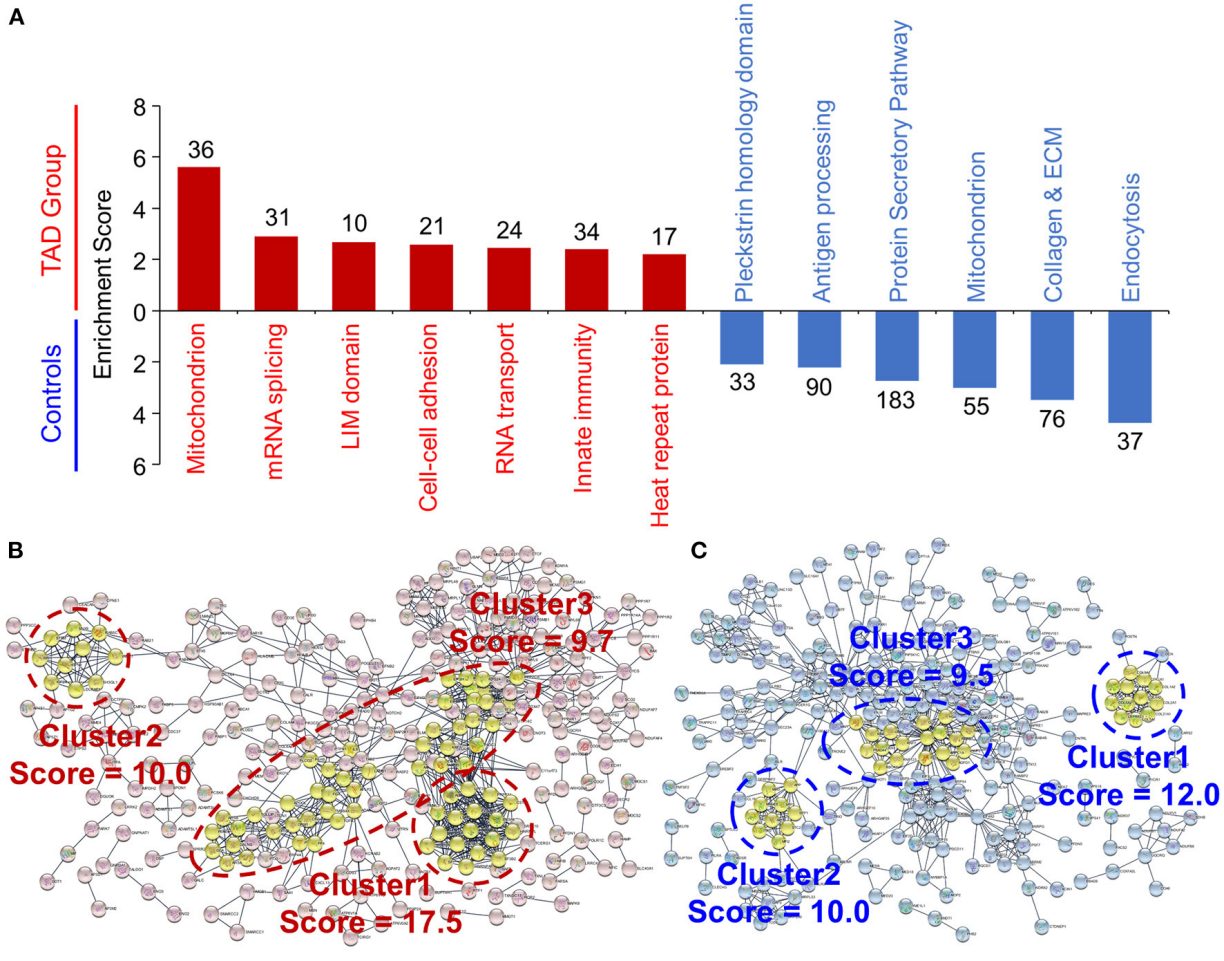
Functional analysis of the differentially expressed proteins in TAD. **(A)** Functional annotation clustering of the differentially expressed proteins. The annotations were analyzed via the Database for Annotation, Visualization and Integrated Discovery (DAVID). All the proteins with an enrichment score over 2.0 are shown. The enriched protein counts of each annotation are provided. The results of the KEGG pathway and GO analyses are shown in Supplementary Figure 2 Protein–protein interaction networks of the upregulated proteins **(B)** and the downregulated proteins **(C)** in TAD samples. The networks were analyzed by STRING and visualized with Cytoscape software. The functional clusters were defined via the Molecular Complex DEtection (MCODE) plugin, and the top three clusters are shown.

Functional annotation enrichment analysis (Database for Annotation, Visualization and Integrated Discovery; DAVID) revealed seven functional clusters that were upregulated in TAD, namely, mitochondrion, mRNA splicing, LIM domain, cell-cell adhesion, RNA transport, innate immunity, and heat repeat protein (Figure 3A), and six functional clusters that were downregulated in TAD, including the annotations in endocytosis, collagen and ECM, mitochondrion, protein secretory pathway, antigen processing and Pleckstrin homology domain. These clusters were consistent with the Kyoto Encyclopedia of Genes and Genomes (KEGG) pathway analysis and Gene Ontology (GO) term analysis (Supplementary Figure 2).

GO term analysis of biological processes revealed a significant enrichment of negative regulation of supramolecular fiber organization in TAD (Supplementary Figure 2B). Moreover, the GO cellular component annotations including collagen trimer, fibrillar collagen trimer, banded collagen fibril, and complex of collagen trimers (Supplementary Figure 2C), and the GO molecular functions annotation of extracellular matrix structural constituent conferring tensile strength (Supplementary Figure 2D) were significantly downregulated in TAD. Consistent with these results, the protein–protein interaction network (STRING) revealed that twelve collagen subunits were enriched in the first cluster of the downregulated proteins (Figures 3B,C). These results suggest that ECM and collagen components are downregulated significantly in aortic tissues from TAD patients.

**Figure 4:**
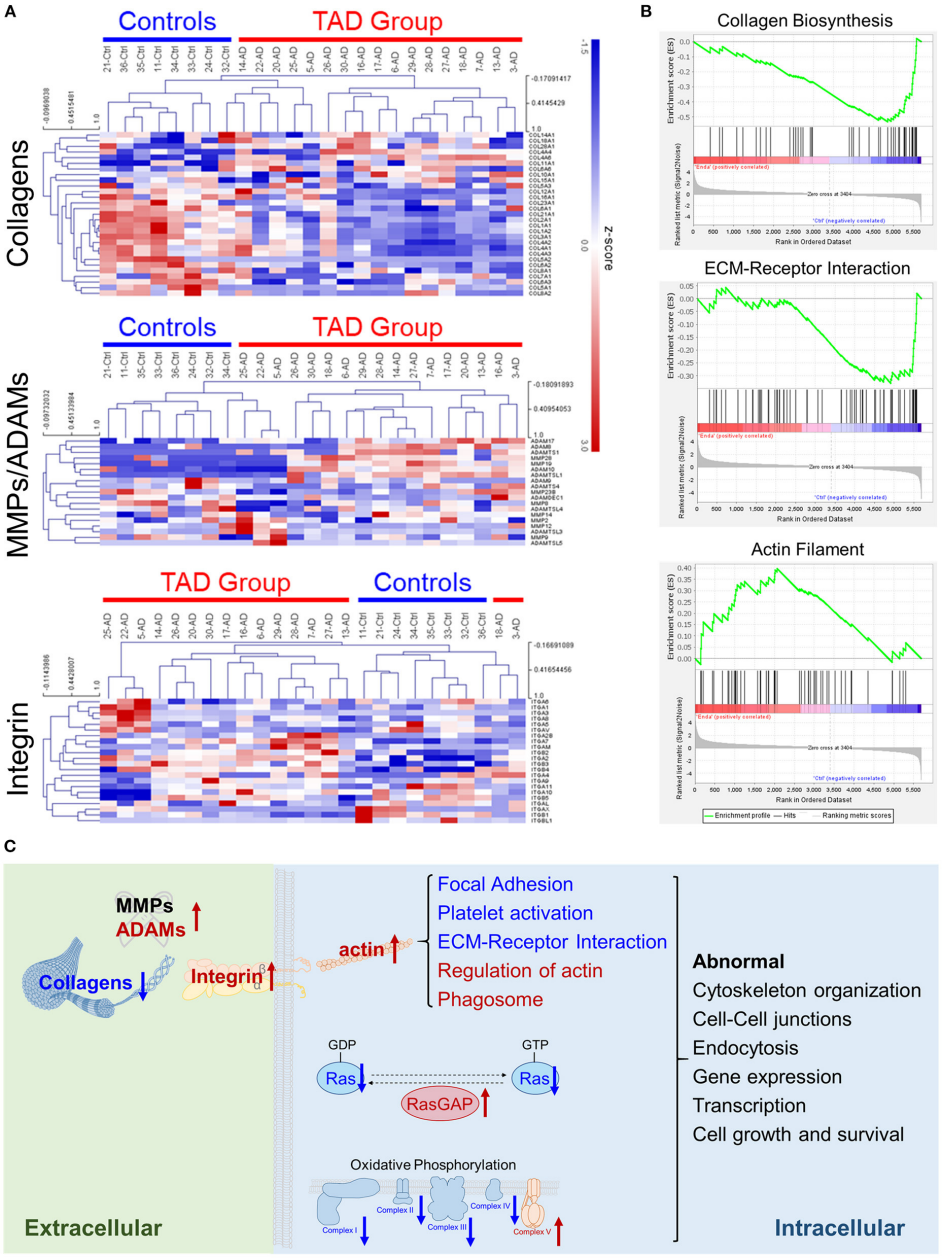
Extracellular matrix (ECM) components are abnormal in TAD. **(A)** Cluster heatmaps of the protein families of collagens, MMPs and ADAMs, or integrins detected in tissue samples. Samples are clustered according to Pearson's correlations. **(B)** Collagen biosynthesis, ECM-receptor interaction, and actin filament were enriched in the gene set enrichment analysis (GSEA). **(C)** Scheme of the abnormally expressed ECM components and pathways in TAD. The upregulated proteins are colored red, and the downregulated proteins are colored blue. The abnormal pathways of RAS and oxidative phosphorylation are shown in detail in Supplementary Figure 3.

Collagens are one of the most dominant components of vascular walls and play essential roles in aortic wall strength (33, 34). Collagen subunits can be processed by extracellular proteases, e.g., matrix metalloproteinases (MMPs) (35), and collagens can interact with cell membrane proteins for cellular signal transduction, e.g., binding with integrins (36). Our proteomics analysis revealed that collagen subunits were significantly downregulated in TAD, and many MMPs and integrins were upregulated (Figure 4A). This abnormal expression might result in the downregulation of ECM-receptor interactions and the upregulation of actin filaments (Figure 4B). The latter is closely associated with many processes, such as focal adhesion and platelet activation, which were abnormal in TAD samples (Figure 3A; Supplementary Figure 2). Moreover, the Ras signaling pathway and oxidative phosphorylation, which are important for cell growth and survival, were also significantly abnormal (Figure 4C). Ras signaling was downregulated, the Ras protein was downregulated, and its negative regulator RasGAP was upregulated (Supplementary Figure 3A). Oxidative phosphorylation was abnormal: complexes I to IV were downregulated, but complex V was upregulated (Supplementary Figure 3B). These abnormities might cause the failure of cell growth and survival of aortic vascular wall cells. Taken together, these results suggest that ECM components and intracellular signaling pathways are abnormal in TAD (Figure 4C) and might play roles in the genesis and development of TAD.

### Measurements of Six Selected Proteins With ELISAs in the Training Phase

**Figure 5:**
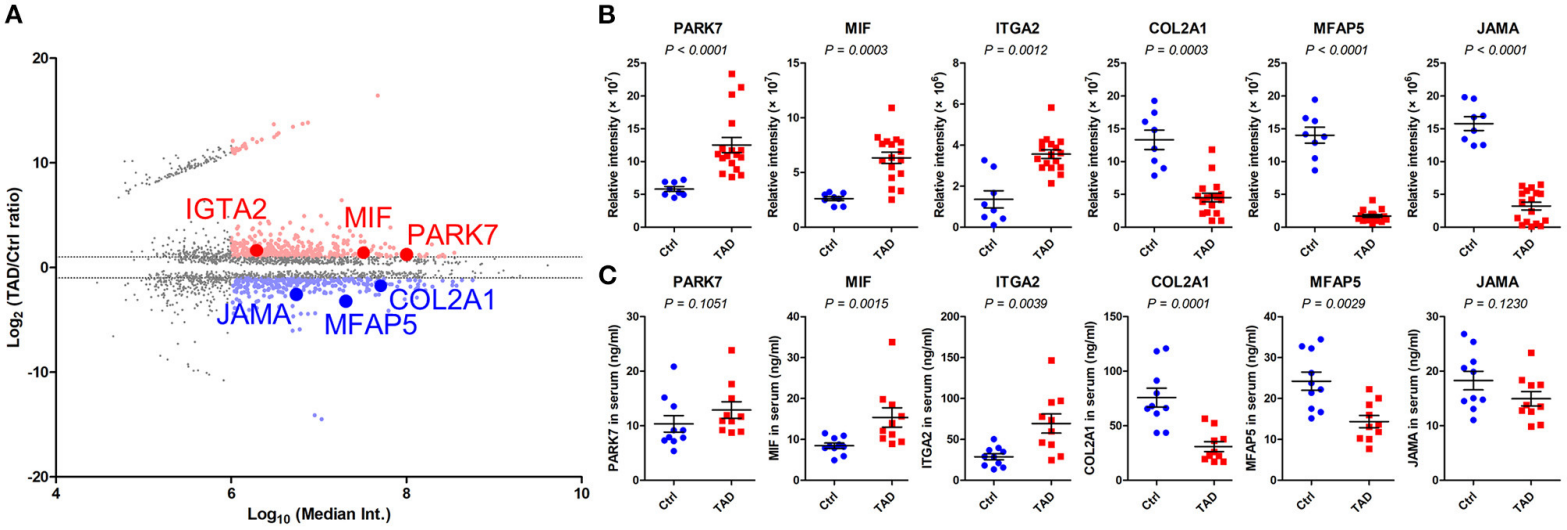
Relative expression of PARK7, MIF, ITGA2, COL2A1, MFAP5, and JAMA in the training phase. **(A)** MA plot of the comparison of the TAD and control proteomes. The proteins with a *P*-value < 0.05 are presented, and those with a median intensity over 1 × 10^6^ are colored red (upregulated) or blue (downregulated). Six selected proteins, PARK7, MIF, ITGA2, COL2A1, MFAP5, and JAMA, were identified. **(B)** Relative expression of the six indicated proteins in tissue samples via proteomics analysis in the screening phase. **(C)** Relative expression of the six indicated proteins in serum samples via ELISAs in the training phase, *n* = 20. *P*-values in **(B,C)** were determined by the Mann-Whitney test.

Based on the criteria described in the study design (Figure 1), six proteins, PARK7, MIF, ITGA2, COL2A1, MFAP5, and JAMA (Figure 5A), were subjected to ELISAs on the serum samples of patients in an independent training set (Table 2). The expression changes in the six proteins in the serum were consistent with those in the proteomics analysis: PARK7, MIF, and ITGA2 were upregulated in TAD, and COL2A1, MFAP5, and JAMA were downregulated in TAD (Figures 5B,C). However, the *P*-values of PARK7 and JAMA in the serum samples were larger than 0.05; therefore, these two proteins were not verified in the training phase. Thus, MIF, ITGA2, COL2A1, and MFAP5 were verified and further evaluated in the validation phase.

**Table 2:** Characteristics of the TAD group and control group in the training phase and validation phase.

**Variable**	**Training phase**			**Validation phase**		
	**TAD (*n* = 10)**	**Control (*n* = 10)**	***P-*value**	**TAD (*n* = 32)**	**Control (*n* = 32)**	***P-*value**
Age (years)	61.10 ± 11.52	76.20 ± 12.43	0.011	65.13 ± 7.38	69.25 ± 10.62	0.076
Male	6 (60%)	9 (90%)	0.121	24 (75%)	24 (75%)	1.000
BMI (kg/m^2^)	22.55 ± 2.85	23.39 ± 3.32	0.553	23.09 ± 4.96	23.69 ± 3.24	0.441
Hypertension	6 (60%)	5 (50%)	0.653	17 (53.1%)	17 (53.1%)	1.000
Diabetes	2 (20%)	5 (50%)	0.160	6 (18.8%)	11 (34.4%)	0.157
Smoker	5 (50%)	6 (60%)	0.653	16 (50%)	16 (50%)	1.000
Marfan syndrome	0	0	1.000	0	0	1.000
Bicuspid aortic valve	0	0	1.000	0	0	1.000
cTnI (ng/ml)	0.04 ± 0.09	0.36 ± 0.11	0.0002	0.13 ± 0.19	0.48 ± 0.22	<0.0001
D-dimer (mg/l)	6.31 ± 10.96	1.14 ± 0.85	0.224	7.79 ± 16.88	1.84 ± 1.77	0.107

*Control group: sreum samples from MI patients. BMI, Body mass index. Relative expression of cTnI and D-dimer was shown in Supplementary Figure 4*.

### Measurements of MIF, ITGA2, COL2A1, and MFAP5 With ELISAs in the Validation Phase

**Figure 6:**
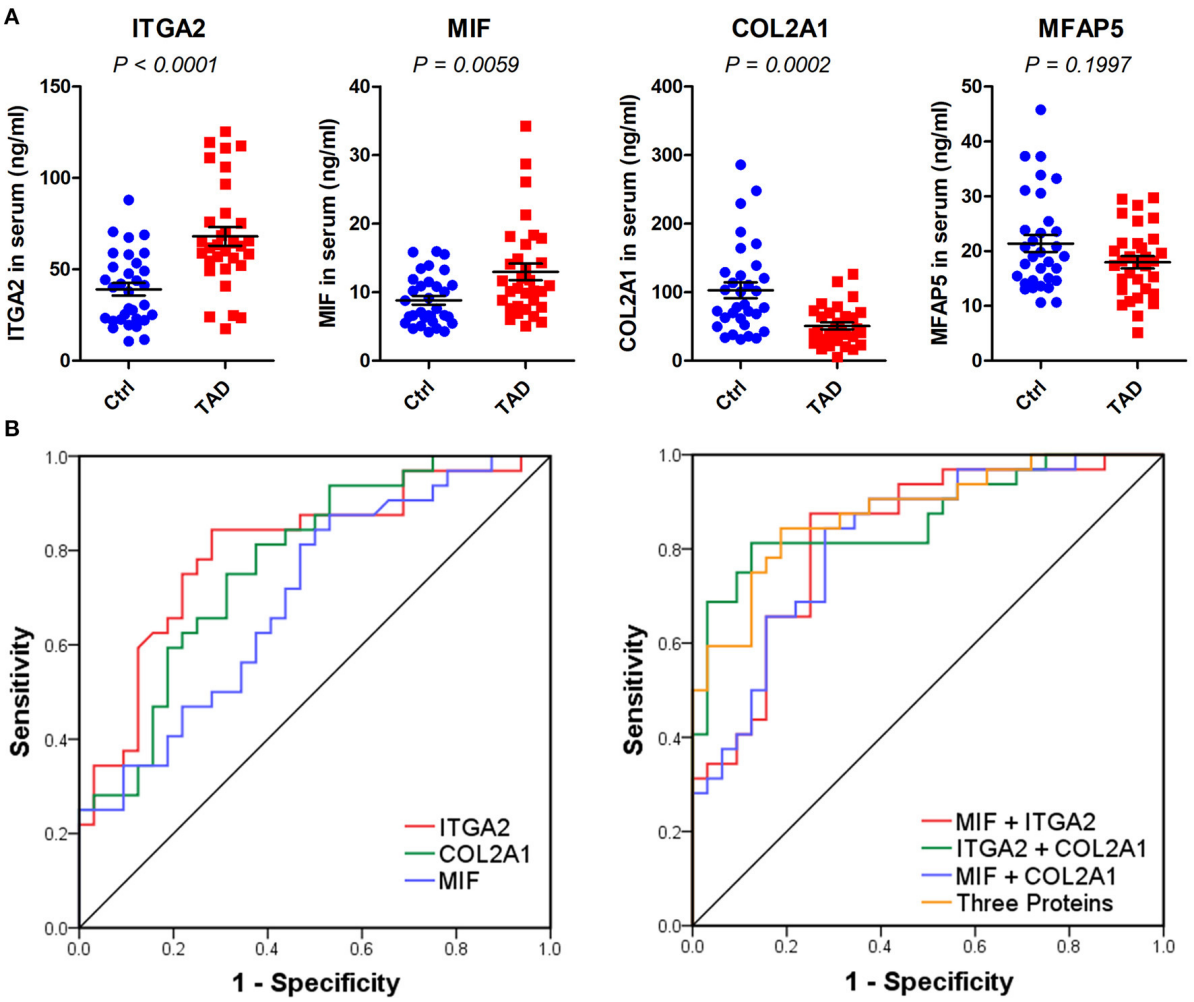
Validation of MIF, ITGA2, COL2A1, and MFAP5 in serum samples via ELISAs. **(A)** Relative expression of the four indicated proteins in the validation phase, *n* = 64. *P*-values were determined by the Mann-Whitney test. **(B)** ROC curves of MIF, ITGA2, COL2A1, and the combination of the three proteins in the validation phase. The predicted values of the combination of each protein were calculated by binary logistic regression.

The four indicated proteins were further analyzed in an independent validation set (Table 2). Three, i.e., MIF, ITGA2, and COL2A1, were verified, with *P*-values of 0.0059, < 0.0001, and 0.0002, respectively (Figure 6A). MFAP5 was not verified, with a *P*-value of 0.1997. We further assessed the diagnostic values of MIF, ITGA2, and COL2A1 in TAD by ROC curve analysis in the validation set. The AUC values of MIF, ITGA2, and COL2A1 were 0.701 (95% CI: 0.574–0.828), 0.801 (95% CI: 0.691–0.911), and 0.773 (95% CI: 0.660–0.887), respectively (Figure 6B). Among these three proteins, ITGA2 showed the best diagnostic power. The AUC values of the combination of each pair of proteins were 0.829 (95% CI: 0.728–0.930) for MIF combined with ITGA2, 0.867 (95% CI: 0.777–0.958) for ITGA2 combined with COL2A1, and 0.822 for MIF combined with COL2A1 (95% CI: 0.720–0.924) (Figure 6B). Moreover, the combination of all three proteins provided a high AUC value of 0.880 (95% CI: 0.727–0.962), with a specificity of 81.2% and a sensitivity of 84.4% (Table 3). Given that age is one of the risk factors for TAD, we also tried to add age as a factor into the ROC curve analysis. As shown in Supplementary Figure 5, combination with age could further improve the AUC values.

**Table 3:** ROC curve analysis of the diagnostic ability of MIF, ITGA2, and COL2A1 in the validation phase.

**Protein**	**AUC**	**95% CI**	**Sensitivity**	**Specificity**	***P*-value**
MIF	0.701	0.574–0.828	81.3%	53.1%	0.006
ITGA2	0.801	0.691–0.911	84.4%	71.9%	<0.001
COL2A1	0.773	0.660–0.887	81.3%	62.5%	<0.001
MIF and ITGA2 panel	0.829	0.728–0.930	87.5%	75.0%	<0.001
ITGA2 and COL2A1 panel	0.867	0.777–0.958	81.3%	87.5%	<0.001
MIF and COL2A1 panel	0.822	0.720–0.924	84.4%	71.9%	<0.001
Three-protein panel	0.880	0.727–0.962	84.4%	81.2%	<0.001

## Discussion

Currently, there is a lack of specific non-invasive biomarkers for the diagnosis of TAD in emergency triage. In this study, we profiled the proteome of aortic tissues from TAD and MI patients and verified three proteins as potential serum biomarkers for TAD. A total of 1,141 differentially expressed proteins were identified. Functional analysis revealed that ECM components, including collagens, ECM proteases, and integrins, and related intracellular signaling pathways were aberrant in TAD. Then, we validated the potential clinical value of ITGA2, COL2A1, MIF, and their combination to diagnose TAD. The findings of our study may have implications in the diagnosis and treatment of TAD.

Collagens are one of the dominant components of vessel walls and are mainly synthesized and secreted by endothelial cells and smooth muscle cells. Among the collagen family, type I and type III collagens are abundant in vessel walls (34). We found that the levels of almost all collagen subunits were reduced significantly in TAD tissues, consistent with previous studies showing that total collagen levels were reduced in tissues with aortic dissections (37). The secreted subunit of collagen II (COL2A1), which displayed a marked decrease in the serum samples of TAD patients, was further validated. The AUC value of COL2A1 (0.773, 95% CI: 0.660–0.887) reveals that COL2A1 is a promising candidate serum biomarker for TAD. Collagens and elastin form elastic fibers at the media of vessel walls, maintaining the physical properties of vessels, e.g., hardness, elasticity, and strength. The thoracic aorta carries the blood that is pumped directly from the heart, which requires more mechanical properties. A massive reduction in collagens would decrease the buffer functions of the aorta to the force of blood pushing against the walls pumped out by the heart, which might increase the risk of TAD. In the ECM, collagen fibers are cleaved and regulated by many proteases, including collagenases, e.g., MMP1 for most types of collagen, and specific proteases, e.g., MMP2 and MMP9 for collagen IV (38, 39). The proteolytic fragments of collagens play roles in the interactions between the ECM and vascular cells. The degraded fragments of type I collagen have been reported to promote the disassembly of smooth muscle cells and focal adhesions (40). Thus, the crosstalk between extracellular proteins and intracellular signal transduction is important for the etiopathology of TAD.

ITGA2, also known as glycoprotein Ia (GP Ia), belongs to the integrin family. It forms a heterodimer with integrin beta 1 (ITGB1) and represents a cell membrane receptor for collagen, laminin, and other ECM proteins (36). The ITGA2/ITGB1 complex is responsible for cell adhesion to collagens and can modulate the expression of collagen and collagenase (36). Our proteomics screening revealed that ITGA2 was significantly upregulated in both the tissue and serum samples of TAD patients. Further ELISAs indicated the good diagnostic capability of ITGA2 to distinguish TAD from MI (AUC value 0.801, 95% CI: 0.691–0.911), suggesting that ITGA2 is a promising serum biomarker for TAD. Consistent with these findings, recent computational biology and bioinformatics studies have revealed that the gene expression of ITGA2 is upregulated in acute type A aortic dissections based on RNA-sequencing data in the Gene Expression Omnibus (GEO) database (41, 42). Moreover, ITGA2 was found to be abnormally expressed in a mouse model of aortic aneurysms (43, 44), another severe aortic disease. This evidence supports that ITGA2 plays an essential role in the etiopathology of aortic diseases, including but not limited to aortic dissection and aneurysms. Several studies also revealed that the genetic polymorphism of ITGA2 was associated with vascular diseases such as coronary atherosclerosis which might cause MI (45, 46). Although the expression of ITGA2 might not change in MI, the genetic polymorphism could be used as a risk factor to predict and prevent MI. Notably, another two integrins, ITGA3 and ITGA5, have been reported to be downregulated in aortic dissections (47). In addition to ITGA2, ITGA3 and ITGA5, which are receptors for laminin and fibronectin, respectively, also associate with ITGB1 to form heterodimers (36). Combined with the different expression levels of ECM proteins in aortic dissections, these results further imply that the abnormal ECM and integrins might be potential postoperative monitoring indicators of TAD.

Inflammation is one of the initiating factors for many vascular diseases. Many studies have revealed that the progressing of aortic dissection is associated with aortic wall inflammation (48). Macrophages, the dominate player for inflammation of arteries, have been reported to play a critical role in aortic dissection (49). Reducing the accumulation of macrophages by indomethacin could decrease the rates of AD in mice model (50). We identified that the macrophage migration inhibitory factor (MIF) was significantly upregulated in both tissues and serum of TAD patients. MIF is a pro-inflammatory cytokine, which could inhibit random migration of monocytes/macrophages (51). MIF has been demonstrated to trigger the dedifferentiation of vascular smooth muscle cells (VSMCs) (52), and the latter is closely related with aortic dissection and dissecting aortic aneurysm (53). Thus, the high expression of MIF in TAD suggested that blockage of MIF may be a therapeutic option for decreasing the inflammations of aorta and preventing the dedifferentiation of VSMCs. Notably, MIF has also been reported as a risk factor in MI (54). The expression of circulating MIF in MI was identified higher than that in healthy control (55). In our study, we found that the serum MIF in TAD was higher than that in MI. It suggested that the inflammation level of TAD might even be higher than that in MI.

Aberrant lipid metabolism is a significant risk factor for cardiovascular disease (56). Apolipoproteins are critical in lipid metabolism for regulating plasma lipid and lipoprotein levels, among which apolipoprotein E (APOE) plays a central role (57). Moreover, in a mouse model of cardiovascular disease, *ApoE* gene knockout greatly improved the success rate of inducing atherosclerosis, aortic aneurysm, and aortic dissections (58, 59). Interestingly, our proteomics study revealed that APOE expression in TAD patient samples was downregulated significantly, with a three-fold decline (Supplementary Figure 6). This result implies that low APOE expression might be one of the risk factors for TAD, and upregulating APOE levels might be a potential targeted treatment for reducing the risk of TAD.

“Chest pain” is one of the common symptoms in the emergency department, and related pathogenic diseases include MI, TAD, pulmonary embolism, and tension pneumothorax, among which the former two have a high mortality rate (60). Due to uneven regional economic development in China, the development level of prehospital emergencies is uneven (60). At present, expensive imaging examinations and biomarkers with poor specificity create a barrier to quickly identifying such high-risk patients. In our previous studies, we attempted to provide clues for the prevention of TADs through simple, inexpensive, and repeatable examinations, such as screening morphological risk factors for TAD in plain chest computed tomography scans (61, 62). And in this study, some potential biomarkers were obtained and great predictive efficacy was achieved. We hope that combining the imaging parameters and biomarkers could establish a multimodal prediction model for the occurrence and prognosis of TAD, and provide a better plan for the prevention and treatment of TAD.

There were still several limitations to this study. First, this study was limited by the sample size. To become applicable biomarkers, more validations on larger sample sizes and additional cohorts from multiple centers are needed. Second, in the screening phase, the factor of age between TAD and control groups was not matched. That was because that the average age of the patients in control group who received CABS in our center was over 65 years, which is the same as the statistical data at the worldwide level (63–65). Although we made the age matching in the validation phase, more validations are needed. Third, although the identified potential biomarkers display good diagnostic value for distinguishing TAD and MI, the disease specificities of these markers should be confirmed. The genetic polymorphism of ITGA2 and the expression of MIF have been reported as risk factors in other vascular diseases. To achieve the aim of guiding the emergency triage, more controls, e.g., patients with other types of severe chest pain and even healthy volunteers, are needed in further validation studies. Fourth, detections with ELISAs are not suitable enough for the clinical application. To be used in clinic, more convenient and stable detection methods, e.g., the chemiluminescence immunoassays, are needed to be developed. Finally, the mechanisms by which ITGA2, COL2A1, and MIF regulate TAD are unclear. We predicted the potential functions and related pathways of these proteins by bioinformatics analysis, but further studies are needed to explore and verify their associations and molecular roles in the etiopathology of TAD.

## Conclusions

We herein report three proteins, ITGA2, COL2A1, and MIF, as novel promising biomarkers for the emergency triage of TAD. The protein families of integrins and collagens were significantly aberrantly expressed in aortic tissues from TAD patients. Crosstalk between the ECM and cell membrane receptors may be a potential postoperative monitoring indicator of TAD. Taken together, our findings suggest the potential value of integrins and collagens in the diagnosis and treatment of TAD. However, this was a pilot study, and further validations in larger sample sizes are needed to evaluate their application value in the clinic.

## Data Availability Statement

The original contributions presented in the study are publicly available. This data can be found here: http://www.ebi.ac.uk/pride, PXD029051.

## Ethics Statement

The studies involving human participants were reviewed and approved by Ethics Committee of Shanghai Ninth People's Hospital, Shanghai Jiao Tong University, School of Medicine. The patients/participants provided their written informed consent to participate in this study. Written informed consent was obtained from the individual(s) for the publication of any potentially identifiable images or data included in this article.

## Author Contributions

PQ, ZX, FC, and XL designed the experiments. PQ, MY, JH, XC, ZWu, QH, SH, YF, ZWe, and BZ collected data and performed experiments for the study. PQ, MY, HP, YY, and ZX analyzed the data. PQ, HP, and ZX wrote the first draft of the paper. CZ, ZX, FC, and XL were responsible for manuscript modification and discussion of the data analysis. All authors contributed to the article and approved the submitted version.

## Funding

This work was supported by the National Natural Science Foundation of China (Nos. 82170509, 22007065, 81900410, 81870346, 81803088, and 81870762), the Open Research Program of National Facility for Translational Medicine (Shanghai) (No. TMSK-2021-121), the Chinese Postdoctoral Science Foundation (No. 2021M692117), Shanghai Science and Technology Innovation Action Plan (Nos. 20Y11909600, 21S31904300), and Shanghai Municipal Health Bureau Project (No. 202040434).

## Conflict of Interest

The authors declare that the research was conducted in the absence of any commercial or financial relationships that could be construed as a potential conflict of interest.

## Publisher's Note

All claims expressed in this article are solely those of the authors and do not necessarily represent those of their affiliated organizations, or those of the publisher, the editors and the reviewers. Any product that may be evaluated in this article, or claim that may be made by its manufacturer, is not guaranteed or endorsed by the publisher.
