# Supplementary material for: Potential Clinical Value of Biomarker-Guided Emergency Triage for Thoracic Aortic Dissection
Source: Front Cardiovasc Med. 2022 Jan 12;8:777327. doi: 10.3389/fcvm.2021.777327 (PMC8790093; doi:10.3389/fcvm.2021.777327)
Supplement: Supplementary file 1 [file Table_1.PDF]

Supplementary Material

Supplementary Table 1 | Individual information of subjects in the screening phase.

Subjects' ID	Group	Age (years)	Gender	BMI (kg/m^2)	Hypertension[a]	Diabetes[a]	Smoker[a]	Marfan syndrome[a]	Bicuspid aortic valve[a]
3	TAD	48	Male	26.53	1	0	1	0	0
5	TAD	59	Female	27.41	1	0	0	0	0
6	TAD	68	Female	24.22	0	0	0	0	0
7	TAD	58	Male	21.48	1	0	1	0	0
13	TAD	30	Male	25.51	0	0	1	0	0
14	TAD	74	Male	25.93	1	0	1	0	0
16	TAD	68	Female	25.89	1	1	0	0	0
17	TAD	53	Male	23.44	1	0	1	0	0
18	TAD	56	Female	22.23	1	0	0	0	0
20	TAD	45	Male	22.31	1	0	1	0	0
22	TAD	56	Male	17.63	1	0	0	0	0
27	TAD	31	Male	21.63	1	0	1	0	0
29	TAD	31	Male	23.88	1	0	1	0	0
30	TAD	49	Male	23.44	1	0	1	0	0
28	TAD	54	Male	20.18	1	0	1	0	0
25	TAD	62	Female	22.86	1	0	0	0	0
26	TAD	65	Female	26.01	0	0	0	0	0
11	Ctrl	57	Male	24.21	1	0	1	0	0
21	Ctrl	72	Male	20.96	1	0	1	0	0
24	Ctrl	75	Male	25.22	0	0	1	0	0
32	Ctrl	78	Male	19.49	0	1	1	0	0
33	Ctrl	72	Female	27.68	1	0	0	0	0
34	Ctrl	67	Male	22.39	1	0	1	0	0
36	Ctrl	66	Female	23.59	0	0	0	0	0
35	Ctrl	60	Female	27.34	1	0	0	0	0

[a] 1, Ture; 0, False.

BMI, Body mass index.

Supplementary Table 2 | Individual information of subjects in the training phase.

Subjects' ID	Group	Age (years)	Gender	BMI (kg/m^2)	Hypertension[a]	Diabetes[a]	Smoker[a]	Marfan syndrome[a]	Bicuspid aortic valve[a]
1	TAD	68	Male	21.13	1	0	1	0	0
2	TAD	73	Female	21.48	1	0	0	0	0
3	TAD	71	Male	25.35	1	1	0	0	0
4	TAD	36	Female	21.63	0	0	0	0	0
5	TAD	59	Male	25.22	1	0	0	0	0
6	TAD	69	Female	19.00	0	1	0	0	0
7	TAD	61	Male	21.48	0	0	1	0	0
8	TAD	48	Male	26.12	1	0	1	0	0
9	TAD	67	Male	25.72	0	0	1	0	0
10	TAD	59	Female	18.37	1	0	1	0	0
11	Ctrl	78	Male	19.49	0	0	0	0	0
12	Ctrl	87	Male	24.22	0	0	1	0	0
13	Ctrl	88	Male	20.52	1	1	1	0	0
14	Ctrl	63	Male	29.41	0	1	1	0	0
15	Ctrl	78	Male	20.05	0	0	0	0	0
16	Ctrl	67	Male	25.06	1	1	0	0	0
17	Ctrl	53	Male	21.22	1	0	0	0	0
18	Ctrl	89	Male	26.67	1	1	1	0	0
19	Ctrl	88	Female	21.48	1	1	1	0	0
20	Ctrl	71	Male	25.75	0	0	1	0	0

[a] 1, Ture; 0, False.

BMI, Body mass index.

Supplementary Table 3 | Individual information of subjects in the validation phase.

Subjects' ID	Group	Age (years)	Gender	BMI (kg/m^2)	Hypertension[a]	Diabetes[a]	Smoker[a]	Marfan syndrome[a]	Bicuspid aortic valve[a]
21	TAD	58	Male	22.66	0	0	0	0	0
22	TAD	73	Male	22.72	1	1	1	0	0
23	TAD	71	Male	28.39	1	0	1	0	0
24	TAD	68	Male	23.59	1	0	1	0	0
25	TAD	76	Male	23.72	1	1	0	0	0
26	TAD	64	Male	19.59	1	1	1	0	0
27	TAD	76	Female	23.44	1	0	0	0	0
28	TAD	69	Male	24.24	1	0	0	0	0
29	TAD	59	Male	21.63	1	0	1	0	0
30	TAD	69	Male	17.30	0	0	1	0	0
31	TAD	78	Female	26.14	1	0	0	0	0
32	TAD	71	Male	21.09	0	0	1	0	0
33	TAD	75	Male	25.00	0	0	0	0	0
34	TAD	69	Male	22.49	0	0	0	0	0
35	TAD	69	Female	17.58	0	0	0	0	0
36	TAD	59	Male	23.15	1	0	1	0	0
37	TAD	66	Male	18.31	0	0	0	0	0
38	TAD	60	Female	24.41	1	1	0	0	0
39	TAD	54	Male	23.66	1	0	1	0	0
40	TAD	63	Male	23.39	0	1	0	0	0
41	TAD	68	Male	19.84	0	0	1	0	0
42	TAD	56	Female	26.31	0	0	1	0	0
43	TAD	74	Male	25.39	1	1	0	0	0
44	TAD	62	Male	20.68	0	0	0	0	0
45	TAD	52	Male	25.65	1	0	1	0	0
46	TAD	52	Male	23.83	0	0	0	0	0
47	TAD	60	Female	22.19	0	0	1	0	0
48	TAD	54	Male	25.25	0	0	1	0	0
49	TAD	61	Female	30.30	1	0	1	0	0
50	TAD	64	Male	21.51	0	0	0	0	0
51	TAD	69	Female	19.53	1	0	1	0	0
52	TAD	65	Male	25.88	1	0	0	0	0
53	Ctrl	72	Male	24.98	0	0	0	0	0
54	Ctrl	70	Male	27.04	1	0	0	0	0
55	Ctrl	64	Female	24.65	0	0	0	0	0
56	Ctrl	80	Male	33.06	0	0	0	0	0
57	Ctrl	57	Male	20.97	0	0	0	0	0
58	Ctrl	81	Male	22.68	1	0	0	0	0
59	Ctrl	61	Male	26.17	1	1	1	0	0
60	Ctrl	83	Male	26.23	0	0	0	0	0
61	Ctrl	69	Male	23.51	1	1	1	0	0
62	Ctrl	61	Male	20.57	1	1	0	0	0

63	Ctrl	66	Male	26.12	1	1	1	0	0
64	Ctrl	77	Male	21.97	1	0	1	0	0
65	Ctrl	73	Female	27.18	1	1	1	0	0
66	Ctrl	82	Male	27.34	1	1	1	0	0
67	Ctrl	69	Male	25.71	1	0	1	0	0
68	Ctrl	63	Male	21.26	1	1	1	0	0
69	Ctrl	67	Male	18.94	1	0	0	0	0
70	Ctrl	65	Male	22.59	1	0	1	0	0
71	Ctrl	77	Female	21.78	0	0	1	0	0
72	Ctrl	58	Female	26.91	0	0	1	0	0
73	Ctrl	59	Male	20.90	0	0	0	0	0
74	Ctrl	74	Male	19.37	1	1	0	0	0
75	Ctrl	78	Male	24.49	1	1	0	0	0
76	Ctrl	63	Male	20.38	0	1	0	0	0
77	Ctrl	90	Female	22.34	1	1	1	0	0
78	Ctrl	68	Male	22.49	1	0	0	0	0
79	Ctrl	61	Male	27.76	0	0	0	0	0
80	Ctrl	64	Female	20.96	0	0	1	0	0
81	Ctrl	67	Female	24.56	0	0	1	0	0
82	Ctrl	79	Female	22.04	0	0	1	0	0
83	Ctrl	35	Male	25.65	0	0	1	0	0
84	Ctrl	83	Male	17.47	0	0	0	0	0

[a] 1, Ture; 0, False.

BMI, Body mass index.
