# Supplementary material for: Potential Clinical Value of Biomarker-Guided Emergency Triage for Thoracic Aortic Dissection
Source: Front Cardiovasc Med. 2022 Jan 12;8:777327. doi: 10.3389/fcvm.2021.777327 (PMC8790093; doi:10.3389/fcvm.2021.777327)
Supplement: Supplementary file 2 [file Image_1.PDF]

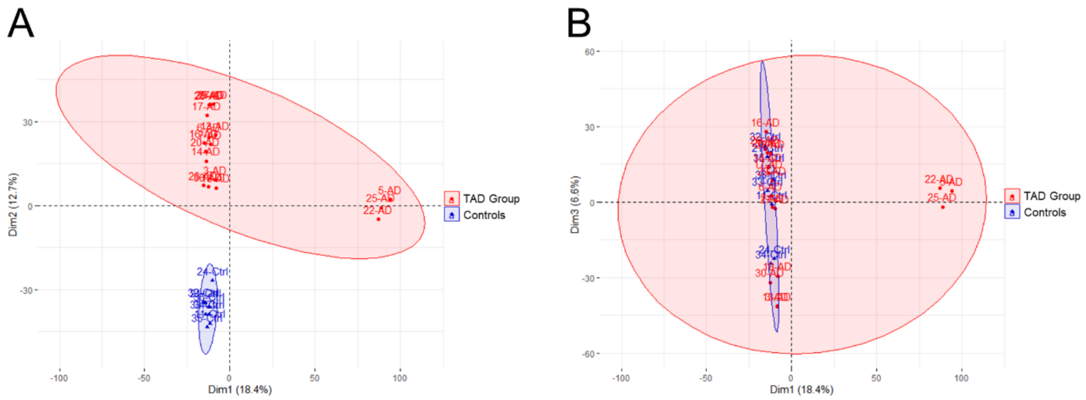

Supplementary Figure 1 | Principal component analysis (PCA) plots of TAD and control samples. Scatter plots show PC1 against PC2 **(A)** and PC1 against PC3 **(B)**.

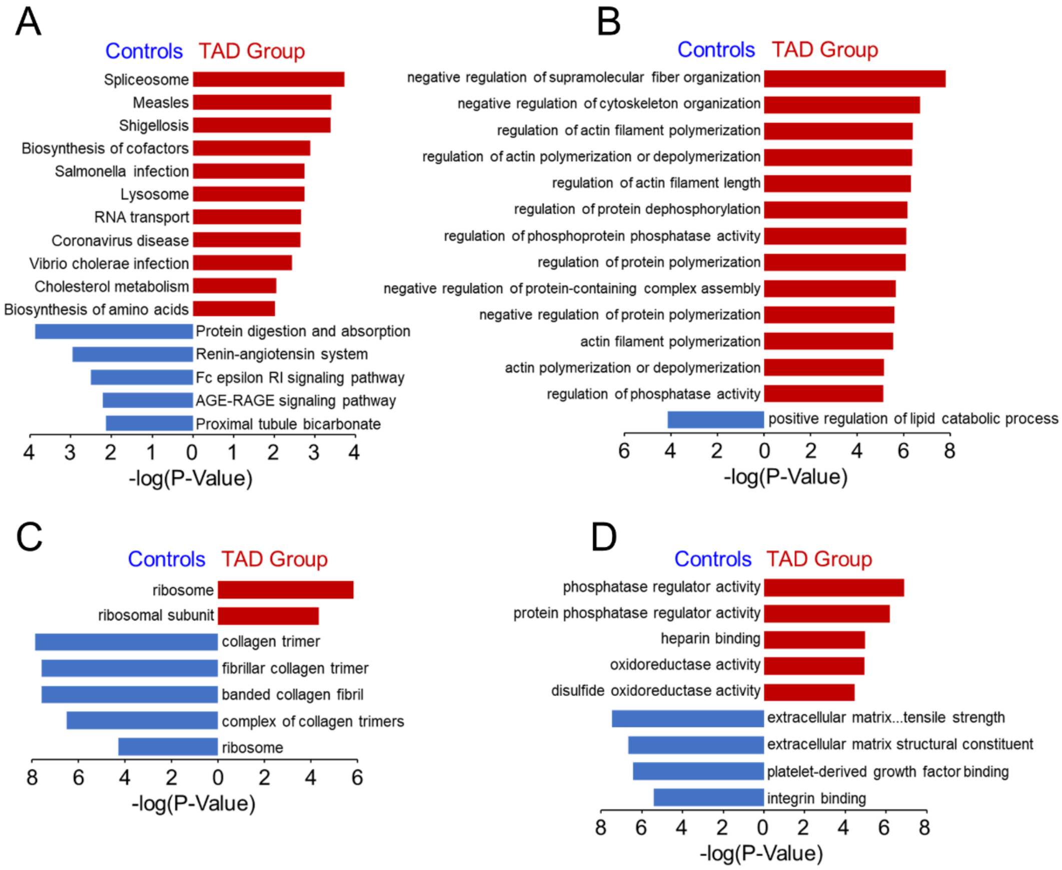

Supplementary Figure 2 | KEGG pathway and GO term analyses of the differentially expressed proteins. **(A)** KEGG pathway analysis. Pathways with a *P*-value < 0.01 are shown. **(B)** GO biological process, **(C)** GO cellular component, and **(D)** GO molecular function. The GO terms with a *P*-value $< 1 \times 10^{-4}$ are shown.

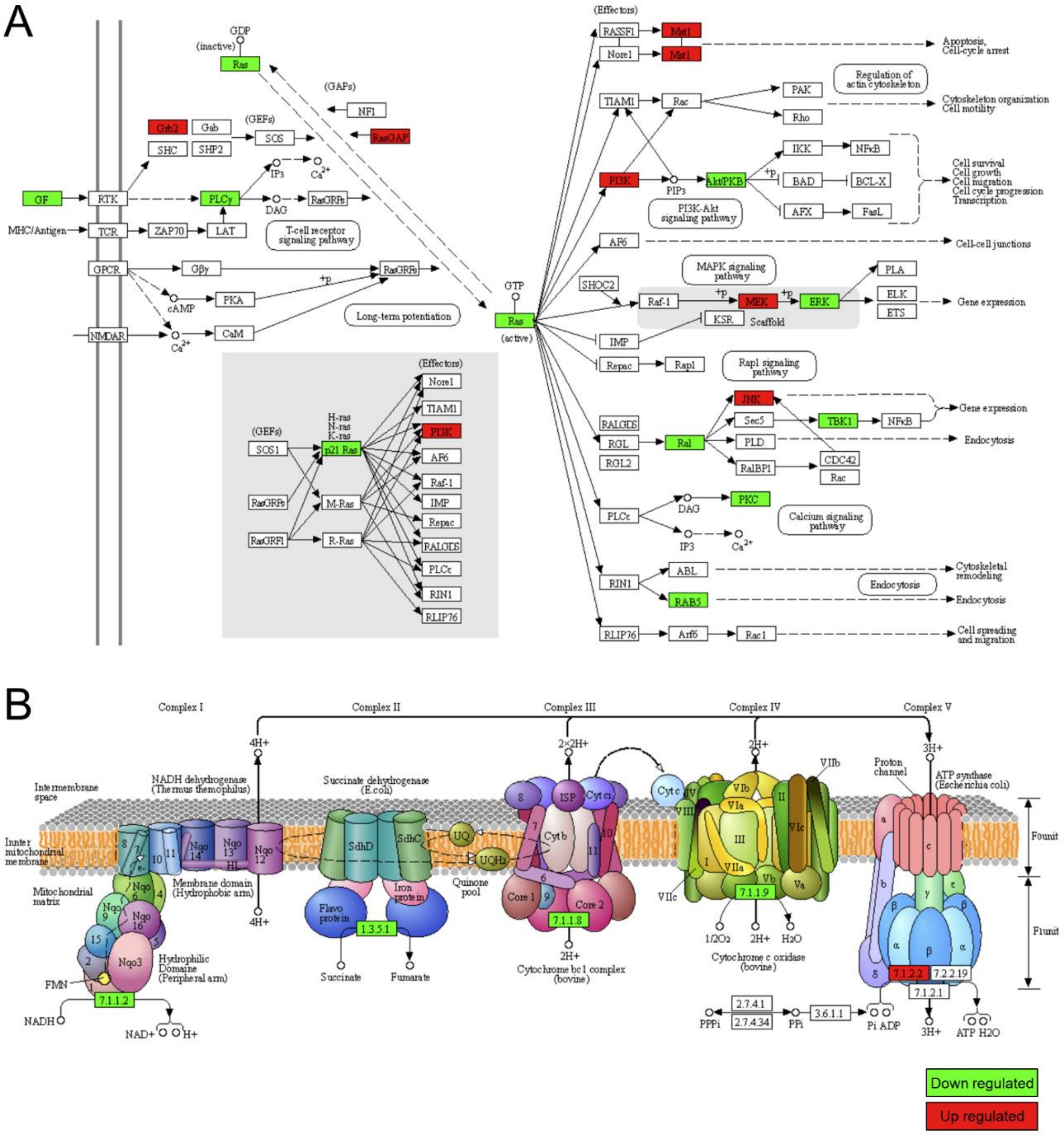

Supplementary Figure 3 | The abnormal pathways: RAS **(A)** and oxidative phosphorylation **(B)**. The pathways were analyzed and visualized by KEGG. The upregulated proteins are colored red, and the downregulated proteins are colored green.

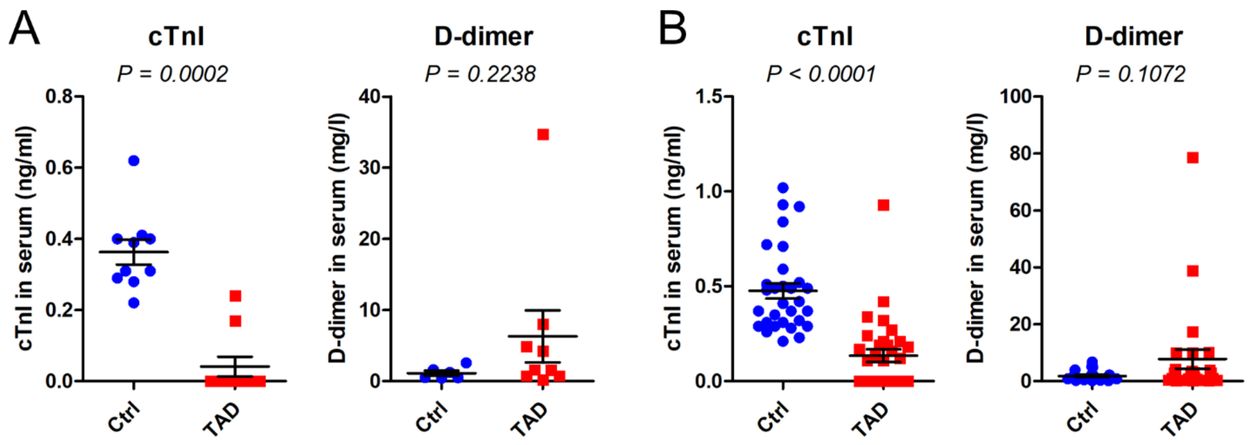

Supplementary Figure 4 | Relative expression of the cTnI and D-dimer in the training phase **(A)** and the validation phase **(B)**. The cTnI was detected by an Beckman Coulter ACCESS2 Immunoassay System. The D-dimer was detected by a Sysmex CS-5100 automatic coagulation instrument.

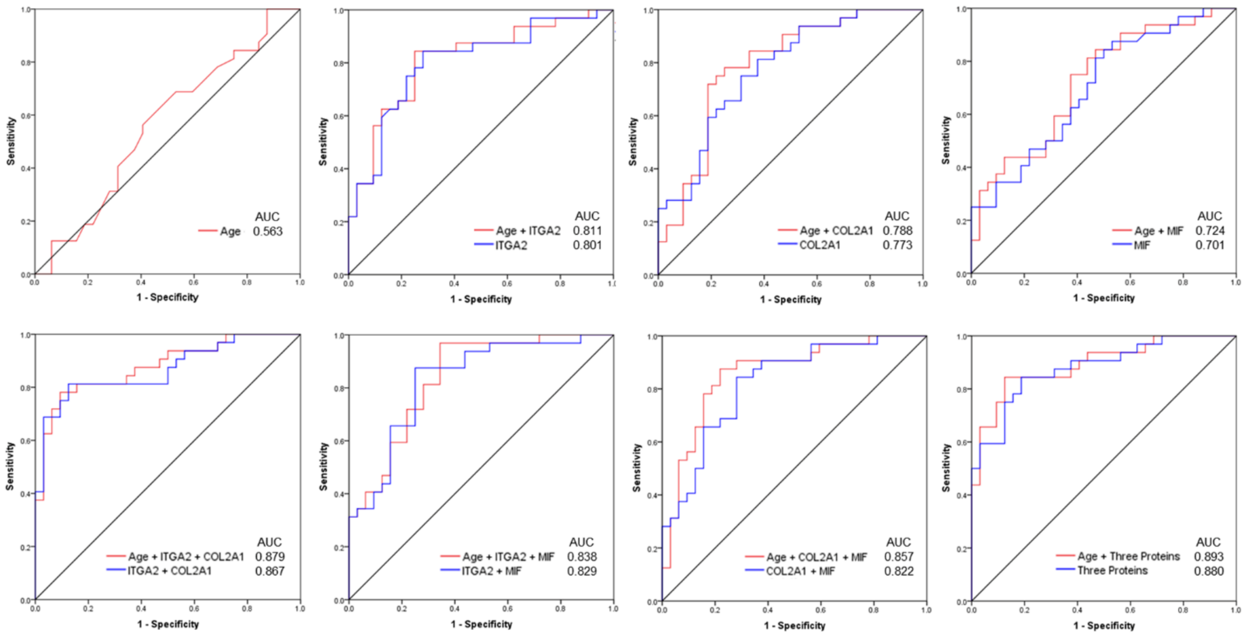

Supplementary Figure 5 | ROC curves of age and the combination of age and MIF, ITGA2, and COL2A1 in the validation phase. The predicted values of the combination of each protein were calculated by binary logistic regression.

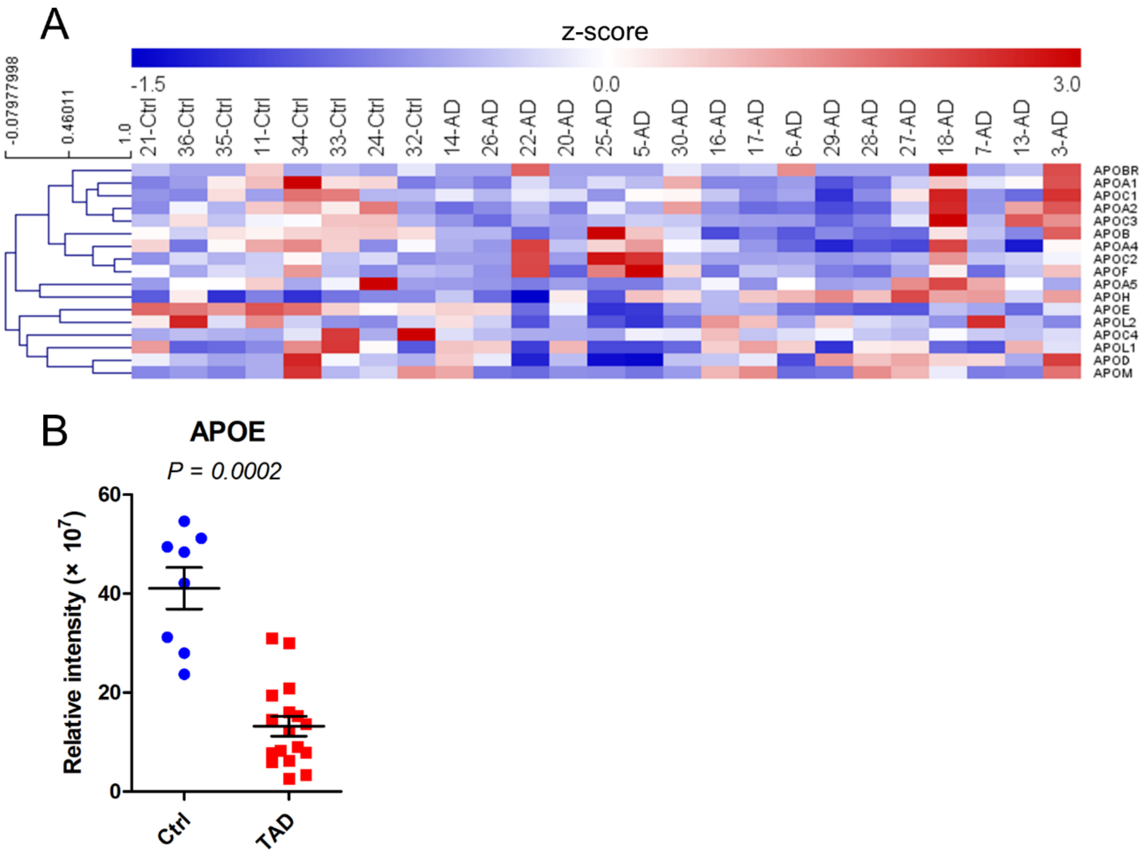

Supplementary Figure 6 | Abnormally expressed lipoproteins in TAD. **(A)** Cluster heatmaps of the detected lipoproteins. **(B)** Relative expression of APOE in the proteomics analysis of TAD and control samples. *P*-values were determined by the Mann-Whitney test.
